# Editorial: Emerging perspectives in sodium channelopathies

**DOI:** 10.3389/fphar.2022.1019004

**Published:** 2022-09-23

**Authors:** J. R. Groome, H. J. Ray, M. Chahine

**Affiliations:** ^1^ Department of Biological Sciences, Idaho State University, Pocatello, ID, United States; ^2^ Department of Medicine, Faculty of Medicine, Université Laval, Quebec City, QC, Canada; ^3^ CERVO Brain Research Center, Quebec City, QC, Canada

**Keywords:** channelopathies, sodium channel, computational modeling, transgenic, genotype to phenotype

Recent advances in molecular biology have greatly increased our understanding of ion channel structure and function. Cloning of genes encoding voltage-gated ion channels expanded the investigation of action potential signaling in neurons and muscle fibers. Gene sequences predicted the crucial role of positively charged residues in S4 segments as voltage sensors. Heterologous expression of channels, with mutagenesis, methane thiosulfonate reagent accessibility, and gating current experiments revealed details of the voltage-gating mechanism in sodium, potassium, and calcium channels. The identification of missense or truncation mutations in genes from patients with disorders of excitability, or channelopathies, led to their functional characterization, and expanded our understanding of the roles of diverse regions in channel function (reviewed by Cannon, 2006). The increasing number of channelopathy mutations identified and characterized as causing mixed gating defects, as well as channelopathies that promote mixed phenotypes, illustrates the challenge in the correlation of genotype to phenotype, and therefore the causality of dysfunction in channelopathy disorders.

Prokaryotic sodium channels have yielded new insights into the mechanisms of channel function, with recent investigations providing detail into mechanisms of drug action and channelopathy dysfunction. Cystallographic and cryo-EM approaches have been increasingly important in the study of eukaryotic sodium channels, providing details of channel structure and enhancing computational approaches such as molecular dynamics. What has become clear is that there is no single mechanism of action for channelopathies and thus no one size fits all treatment for patients. Using diverse approaches to gain further understanding of specific genotype/phenotype relationships will facilitate more effective personalized treatment strategies that can greatly enhance patient outcomes. In this Research Topic issue, research articles on sodium channelopathies highlight advancements in our understanding of diseases of excitability, as well as the challenges inherent in bridging the gap in our knowledge of the relationship of genotype, channel dysfunction and patient phenotype.

The database for channelopathy variants has continued to outpace functional characterization. In two manuscripts in this issue from the laboratories of Kostareva et al., computational approaches highlight molecular modeling to predict and molecular dynamics to test the impact of mutations associated with cardiac arrhythmia syndromes. Intersegmental contacts of significance are investigated to identify interactions that may be critical in the coupling of pore and voltage sensor domains. Residues identified as harboring missense variants causing Brugada or long QT syndromes are screened to suggest contacts from mutations coupled to the same disease and located in regions of known channel function. This computational approach provides a pathogenicity “algorithm” that may augment those based on a bioinformatics approach (Holland et al., 2017). A combined computational and functional approach targets a more limited set of potential contacts that may be crucial to the coupling between the voltage sensor domain III and the domain IV S6 pore loop.

Additional contributions to the sodium channelopathy database include variants in accessory, beta subunits. Functional characterizations of beta subunit variants causal to channelopathy syndromes are few, with notable exceptions as in GEFS+ (Wallace et al., 1998). In this issue, a report from the Angsutararux et al. lab describes how mutations in beta subunits differentially produce cardiac sodium channel dysfunction by modulating channel expression (β1) or gating parameters (β3), using immunofluorescence and voltage clamp fluorometry to distinguish these pathological effects. As more cryo-EM structures are provided, the atomistic detail of beta subunit to alpha subunit interaction is becoming increasingly available and is taken advantage of in this report to compare beta subunit variants associated with atrial fibrillation or Brugada syndrome.

The identification of spontaneous or inherited sodium channel *SCN1A* mutations in patients with epilepsy syndromes and their functional characterization has been followed with a continual expansion of the epilepsy variant database. In this issue, a report from the Jones et al. laboratory describes the functional characterization of a rare domain IV *SCN1A* epilepsy variant associated with frontal lobe epilepsy. The effects of the mutation to produce both gain and loss of function is an example of mixed gating defects once thought atypical for *SCN1A* epilepsy variants and points out the growing number of sodium channelopathy variants in excitable tissue with mixed effects and the importance of including these in models of action potential signaling.

Channelopathy mutations in the skeletal muscle channel *SCN4A* also produce gain of function, loss of function or mixed gating effects, with both a challenge and some interesting unifying theories on mutational cause of specific neuromuscular disorders (i.e. gating pore currents in hypokalemic periodic paralysis). In this issue, the *SCN4A* landscape of loss of function mutations for specific channelopathies such as periodic paralysis and congenital myasthenic syndrome is reviewed (Nicole and Lory). A classification of loss of function disorders as sodium channel weakness is proposed, with the goal to facilitate specific therapeutic interventions. Re-classification of sodium channelopathies is also proposed for cardiac arrhythmia syndromes based on pharmacological profiles specific to access of drugs through fenestrations. Originally described in prokaryotic sodium channels, fenestrations have also been demonstrated in eukaryotic sodium channels. The review provided from El-Din et al. describes their impact on cardiac sodium channel function as well as their significance in our understanding and development of pharmacological intervention in cardiac arrythmia. Interestingly, it was suggested that flecainide, a class 1C antiarrhythmic drug, may enter the inner pore through one of these fenestrations. This was somehow surprising since flecainide is known for its open channel block property (reviewed by O’Leary and Chahine, 2018).

The use of transgenic tissue or animals to study channelopathy disorders has been facilitated with advancement in molecular strategies. An article from Shapiro et al. describes a mouse model of *SCN8A* for which in-frame deletions are matched to those identified in human patients with developmental encephalopathy. A separate report from this laboratory uses the transgenic approach to study the effects of cannabidiol on a point mutation in the *SCN8A* gene, illustrating the use of transgenics to evaluate the suggested potential therapeutic effect of cannabidiol on patients with *SCN8A* epilepsy. These studies highlight the evolution from knock-out or knock-in of entire genes to transgenic approaches that target specific mutations to study behavioral phenotypes and test pharmacological intervention.
